# Comparison of chest X-ray interpretation by Emergency Department clinicians and radiologists in suspected COVID-19 infection: a retrospective cohort study

**DOI:** 10.1259/bjro.20200020

**Published:** 2020-08-28

**Authors:** Oliver J Kemp, Daniel J Watson, Carla L Swanson-Low, James A Cameron, Johannes Von Vopelius-Feldt

**Affiliations:** 1 Southmead Hospital Emergency Department, North Bristol NHS Trust, Bristol, United Kingdom; 2 Emergency Care Research Group, Faculty of Health and Applied Sciences, University of the West of England Bristol, Bristol, United Kingdom

## Abstract

**Objectives:**

We describe the inter-rater agreement between Emergency Department (ED) clinicians and reporting radiologists in the interpretation of chest X-rays (CXRs) in patients presenting to ED with suspected COVID-19.

**Methods:**

We undertook a retrospective cohort study of patients with suspected COVID-19. We compared ED clinicians’ and radiologists’ interpretation of the CXRs according to British Society of Thoracic Imaging (BSTI) guidelines, using the area under the receiver operator curve (ROC area).

**Results:**

CXRs of 152 cases with suspected COVID-19 infection were included. Sensitivity and specificity for ‘classic’ COVID-19 CXR findings reported by ED clinician was 84 and 83%, respectively, with a ROC area of 0.84 (95%CI 0.77 to 0.90). Accuracy improved with ED clinicians’ experience, with ROC areas of 0.73 (95%CI 0.45 to 1.00), 0.81 (95%CI 0.73 to 0.89), 1.00 (95%CI 1.00 to 1.00) and 0.90 (95%CI 0.70 to 1.00) for foundation year doctors, senior house officers, higher speciality trainees and ED consultants, respectively (*p* < 0.001).

**Conclusions:**

ED clinicians demonstrated moderate inter-rater agreement with reporting radiologists according to the BSTI COVID-19 classifications. The improvement in accuracy with ED clinician experience suggests training of junior ED clinicians in the interpretation of COVID-19 related CXRs might be beneficial. Large-scale survey studies might be useful in the further evaluation of this topic.

**Advances in knowledge:**

This is the first study to examine inter-rater agreement between ED clinicians and radiologists in regards to COVID-19 CXR interpretation.

Further service configurations such as 24-hr hot reporting of CXRs can be guided by these data, as well as an ongoing, nationwide follow-up study.

## Introduction

COVID-19 is the disease response to the severe acute respiratory syndrome coronavirus 2 (SARS-CoV-2).^1^ As of 5 May 2020, there were over 3.5 million confirmed cases and 243,401 deaths due to this condition worldwide.^2^ Accurate diagnosis of COVID-19 in the Emergency Department (ED) is important as it affects immediate treatment and management both for the individual patient and the wider hospital system.^3^ Clinical features of COVID-19 are non-specific and estimates of the sensitivity of oral-pharyngeal swabs in COVID-19 patients are 60–73.3%.^4,5^ In the ED, the chest radiograph (CXR) is an important early screening tool and guidelines of the British Society for Thoracic Imaging (BSTI) recommend CXRs as the primary imaging modality.^6^ ED clinicians need to reliably recognise classic COVID-19 CXR signs, as well as differentiate these from other important pathologies which require specific treatment. This study describes the accuracy of ED clinicians’ CXR interpretation in cases of suspected COVID-19 infection, when compared to radiologist opinion.

## Methods

We undertook a retrospective cohort study at a single ED in Southwest England (Southmead Hospital, North Bristol NHS Trust) between March and April 2020. Included in this study were patients who presented to the local ED and fulfilled both of the following inclusion criteria:

- Adult patient triaged to the COVID-19 Assessment area due to either pyrexia, shortness of breath or a new cough.
- COVID-19 considered to be the most likely diagnosis by the treating clinician

Patients were included in the study consecutively throughout the study period. Data obtained for this research included the treating ED clinician’s interpretation of the patient’s CXR as well as the formal radiology report, both according to the BSTI COVID-19 guidelines.^7^ The guidelines define ‘*Classic or Probable COVID-19’* findings as *‘predominantly lower lobe and peripheral opacities that are multiple and bilateral’* (see reference for examples images).^6^ All CXRs were reported by higher specialty radiology trainees (more than 3 years in training) or consultant radiologists. We excluded cases where the radiology report was available prior to submission of data by the ED clinician. Entries from ED clinicians with missing data were excluded from the study. All patients who were admitted to hospital had nasopharyngeal swabs taken for reverse transcription polymerase chain reaction testing for COVID-19.

We examined inter-rater agreeability between treating ED clinicians and reporting radiologists using area under the receiver operating curve (ROC area) for a binary ‘classic COVID-19’ *vs* ‘other’ classification. ROC areas were compared using the χ^2^ test.^8^ The need for research ethics committee review was waived by the Health Research Authority based on the fact that only anonymised data were obtained for a COVID-19 research project, from a locally authorised clinical effectiveness project (North Bristol NHS Trust reference number CE44619).^9^ Researchers involved in data analysis were excluded from data collection.

## Results

Between 26 March and 28 April 2020, a convenient sample size of 152 cases with suspected COVID-19 infection and CXRs fit the inclusion criteria, 4 cases were removed due to missing data. The median age was 59 years with 72 female and 80 male patients. Of the 152 cases, 127 were admitted to hospital and 25 were discharged from ED. 59 of the cases subsequently tested positive by RT-PCR, 65 were negative and 28 were not swabbed. Of the 59 cases in which the swabs returned as positive; 34 were reported as *‘Classic/Probable COVID-19’* by the radiologist, 11 as *‘Indeterminate’*, 10 as ‘*Normal’* and 4 as ‘*Non-COVID-19’*. In 16 cases, the radiologist reported the CXR as *‘Classic/Probable’* but the RT-PCR was subsequently negative.

The overall mortality rate was 8.6% (13/152). The CXRs were interpreted by 49 ED clinicians of differing seniority, including 32 non-ED trainee doctors and 17 ED specialty trainees/consultants. Table 1 shows ED clinicians’ categorisation of CXRs compared to the reporting radiologists.

**Table 1.** Comparison of ED clinician and Radiologist categorisation of chest radiographs according to BSTI guidelines

ED clinicians	Reporting radiologists				
	Normal	Classic/ Probable COVID-19	Indeterminate for COVID-19	Non-COVID-19	Total
Normal	38 (83%)	1 (2%)	5 (11%)	2 (4%)	**46**
Classic/ Probable COVID-19	4 (7%)	42 (71%)	10 (17%)	3 (5%)	**59**
Indeterminate for COVID-19	13 (34%)	6 (16%)	15 (39%)	4 (11%)	**38**
Non-COVID-19	3 (33%)	1 (11%)	4 (44%)	1 (11%)	**9**
**Total**	**58**	**50**	**34**	**10**	**152**

BSTI, British Society of Thoracic Imaging; ED, emergency department.

Shaded areas represent agreement between ED clinicians and reporting radiologists.

Overall sensitivity and specificity for *‘Classic or Probable COVID-19’* CXR findings reported by ED clinician was 84 and 83%, respectively, with a ROC area of 0.84 (95%CI 0.77 to 0.90). Accuracy improved with ED clinicians’ experience, with ROC areas of 0.73 (95%CI 0.45 to 1.00), 0.81 (95%CI 0.73 to 0.89), 1.00 (95%CI 1.00 to 1.00) and 0.90 (95%CI 0.70 to 1.00) for foundation year doctors, senior house officers, higher speciality trainees and ED consultants, respectively (*p* < 0.001).

## Discussion

In this analysis of 152 CXRs, ED clinicians demonstrated moderate sensitivity and specificity in recognising *‘Classic/Probable COVID-19’* findings on CXR, when compared to formal radiology reporting.

There are several potential causes for these findings. First, it is important to acknowledge the inherent uncertainty of CXR interpretation, with significant disagreement amongst experienced radiologists reported as high as 11–19%.^10^ Second, these data were captured during the early phase of the first UK COVID-19 peak. ED clinicians were unlikely to have had prior experience in diagnosing COVID-19 CXR changes or prior formal teaching in this area. Within the context of background uncertainty and relatively new pathology, the accuracy with which ED clinicians in this study identified classic COVID-19 signs can be interpreted as reassuring.

ED clinicians were more likely to label CXRs as *‘Classic/Probable COVID-19’* compared to radiologists, whereas radiologists more often described CXRs as ‘*Normal’* compared to ED clinicians. This might suggest a tendency for ED clinicians to overdiagnose COVID-19 when interpreting CXRs, or it could be due to the additional clinical information available to the ED clinician which might influence interpretation. Knowledge of pertinent clinical information has been shown to significantly increase the accuracy of CXR interpretation by radiologists.^11^ The moderate inter-rater agreement between ED clinicians and radiologists for the BSTI COVID-19 classifications in our study highlights potential quality improvement interventions aimed at improving clinical information sharing. Likewise, our data suggest that training of junior ED clinicians in the interpretation of COVID-19 related CXRs might be beneficial to increase overall accuracy.

At the peak of the COVID-19 pandemic in the UK, most UK radiology departments have been able to provide 24-hr hot-reporting of ED CXRs, with considerable implications on resource utilisation. As the UK is now moving to a containment phase with less frequent but ongoing COVID-19 presentations, accurate data on the need for ongoing hot-reporting can support decision-making and resource allocation.

Given the overall relatively low sensitivity of CXRs in identifying COVID-19 patients when compared to CT scans,^12^ other imaging modalities, such as ultrasound, or new machine learning algorithms have gained considerable interest.^13,14^ However, ultrasound frequently suffers from issues of inter-rater reliability and neither ultrasound nor machine learning algorithms have been rigorously tested.^13,14^ While these are promising technologies, our study provides important baseline data for the currently most frequently used imaging modality in the UK.^6^

Limitations of this study include the single-centre retrospective observational research design. Due to the observational nature of the study, only very few patients underwent CT scans, which would probably be considered the gold-standard of diagnosis for COVID-19 pneumonia. Large-scale survey studies might be useful in the further evaluation of this topic.
